# A Novel Approach to Improve the Microstructure and Mechanical Properties of Al–Mg–Si Aluminum Alloys during Twin-Roll Casting

**DOI:** 10.3390/ma13071713

**Published:** 2020-04-06

**Authors:** Yong Li, Chen He, Jiadong Li, Zhaodong Wang, Di Wu, Guangming Xu

**Affiliations:** 1 State Key Laboratory of Rolling and Automation, Northeastern University, Shenyang 110819, China; liyong.neu@vip.163.com (Y.L.); lijd@ral.neu.edu.cn (J.L.); zhdwang@mail.neu.edu.cn (Z.W.); wudi@mail.neu.edu.cn (D.W.); 2 Key Laboratory of Electromagnetic Processing of Materials, Ministry of Education, Northeastern University, Shenyang 110819, China; xu_gm@epm.neu.edu.cn

**Keywords:** twin-roll casting, Al–Mg–Si alloy, forced-cooling, segregation, mechanical properties

## Abstract

The main purpose of this present study was to investigate the different processing conditions on the microstructure, segregation behavior of alloying elements, and mechanical properties of Al−Mg−Si alloy twin-roll cast slab prepared using a novel twin-roll casting technology. The simulation of temperature field, distribution of alloying elements, tensile properties, hardness, and conductivity were examined by a Leica optical microscope, scanning electron microscopy, energy dispersion spectroscopy, electron probe microanalysis, and tensile tests. The results indicated that when the traditional twin-roll casting method was used to produce aluminum alloy strip, there are obvious centerline segregation defects due to the deep crystallization front depth and symmetrical solidification characteristics. When the forced-cooling technology was applied in the twin-roll casting process, by virtue of the changing of crystallization front depth and crystallization front shape, the segregation defects are obviously suppressed. Suggesting that this method can significantly improve the uniformity of alloying elements in the thickness direction of the twin-roll cast slab, ultimately improve the mechanical properties of AA6022 aluminum alloy.

## 1. Introduction

Al−Mg−Si series aluminum alloy is widely used in the field of transportation due to its superior comprehensive properties, such as low density, good stamping performance, high surface quality, good corrosion resistance, and easy recycling [1,2]. The conventional route of producing rolling slabs is direct chilling semi-continuous casting followed by homogenization, surface scalping, hot/cold-rolling, intermediate annealing, and heat treatment, which has the disadvantage of complex process, high cost, and material consumption [3]. Twin-roll casting technology can produce strips directly from melt and thus has the advantage of short process, energy saving, low cost, and better properties. However, it soon became obvious that the technology introduced some features in the finished product that limited its use compared with conventionally produced materials. The most serious disadvantage is that when the aluminum alloy with high alloy element content is produced by twin-roll casting method, because of its wide solidification range, the centerline segregation defects easily occurred in the casting rolling strip, which makes the twin-roll cast products crack in the subsequent processing [4]. Therefore, it is urgent to develop a novel method to produce aluminum alloy of wide solidification range with fewer segregation defects and better properties, which has the prospect of engineering application.

During twin-roll casting (TRC) of aluminum alloy, process parameters such as pouring temperature, cast rolling speed, water cooling, and side dam decide whether the sheet can be successfully prepared as well as the microstructure of the sheet, the heterogeneity of alloying elements is a typical and inevitable feature in twin-roll casting process [5,6]. Previous research of the formation mechanism of surface bleeds and centerline segregation during the twin-roll casting of aluminum and magnesium alloys indicated that the centerline macro-segregation of solute elements is a serious aspect to limit the performance of rolling sheet and can significantly affect the mechanical properties, which need to be solved urgently [7,8,9,10]. Recently, many researchers have successfully fabricated aluminum alloy strips using twin-roll casting technology with a copper roller sleeve, effect of roll casting speed, roll gap, cooling water flow rate, initial cooling water temperature, and initial temperatures of the melt were considered, and the results indicated that homogeneous microstructures and improved mechanical properties were obtained by increasing the cast rolling speed and roll gap thickness [11,12,13]. 

However, there is a certain effect on the reduction of segregation defects, which cannot be completely eliminated. Because of the rolling effect, under certain conditions, the melt rich in alloying elements fluid flowed along the dendrite gap and finally solidified on the surface of the strip to form surface segregation defects.

In addition, to change the conventional processing parameters, a new method of static magnetic field, pulse electric current field, oscillating field, and electromagnetic oscillation field to control micro and macro-segregation of high alloy content aluminum alloys during twin-roll casting process have been reported. The mechanism of external field reduced the formation of segregation defects by controlling the atomic migration of solute elements [14,15,16,17,18,19]. The melt-condition experiments also reported that macroscopic mechanical stirring can also significantly reduce the centerline segregation, this stirring effect can disintegrate and disperse oxide film particles acting as nucleating sites for matrix metal, a fine uniform equiaxed structure would form, avoiding the formation of large area macro-segregation [20,21,22,23]. Due to the inherent solidification characteristics of twin-roll casting technology, segregation defects are unavoidable, but we can study the solidification behavior and controlling the solidification process of alloys to minimize segregation defects as much as possible. Birol et al. investigated the solidification curves of several popular aluminum alloys by the differential scanning calorimetry (DSC) method to identify its impact on the occurrence of macro segregation tendency, and the macro segregation are in full agreement with the predictions from the analysis of solidification curves [24]. 

To the best of our knowledge, a lot of experiments and simulations have been performed to study the effect of traditional processing parameters on the element distribution, mechanical properties, and the formation mechanism of segregation defects. However, no attempt has been made to prepare a twin-roll cast thin slab with high alloy content and wide solidification range by a novel twin-roll casting technology. To fill this gap, the microstructure and mechanical properties of the twin-roll cast thin slabs produced by this novel method with different processing conditions were investigated in this present work, finally readily available for possible automotive industrial applications.

## 2. Materials and Methods

**Figure 1:**
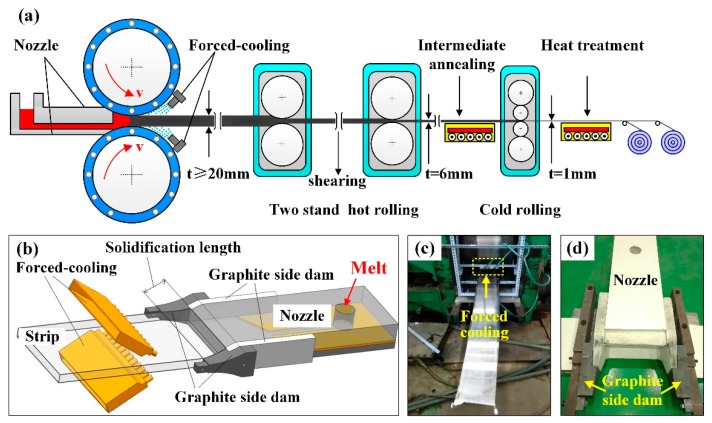
Schematic of twin-roll casting process with forced-cooling (FC-TRC) (**a**). Forced-cooling technology, (**b**) position of forced-cooling device (**c**), and surface quality of twin-roll cast slab (**d**).

As shown in Figure 1, a horizontal twin-roll caster (Northeastern University, Shenyang, China) was adopted in this study and the horizontal caster with two rolls 500 mm in diameter and 500 mm in width. The casting roll is composed of a sleeve and a core with many channels for cooling water inside, which can quickly transfer the heat and reduce the temperature of the roller sleeve. The thickness of the roll sleeve, maximum roll gap, separation rolling force, and cooling water pressure are 50 mm, 50 mm, 1000 kN, and 0.4 MPa, respectively. 

As shown in Figure 1, this is the schematic of twin-roll casting process with forced-cooling and the experimental equipment were independently developed by Northeastern University. In order to fabricate the thin slab with higher quality surfaces, minimum centerline segregations and better mechanical properties, a new graphite side dam (Figure 1d) and forced-cooling device (Figure 1c) was designed in this paper and the graphite side dam is made of high-purity graphite, the secondary forced-cooling medium is water, and the cooling water pressure is 0.4 MPa. The new nozzle is based on the original design of the casting nozzle, replacing the front of the side dam with graphite (Figure 1d), so as to ensure that the casting nozzle will not be damaged during the forced cooling process and provide enough cooling effect for the melt of aluminum alloy in the cast-rolling zone.

The chemical composition (in wt. %) of AA6022 aluminum alloy is Si 1.0 wt. %, Mg 0.8 wt. %, Cu 0.1 wt. %, Mn 0.1 wt. %, Fe 0.1 wt. %, Ce 0.15 wt. %, while the balance is Al. The aluminum alloys were melted in a resistance furnace with TiO_2_ coated stainless steel crucible, and held at 720 °C for three hours, to ensure the alloying elements completely dissolved and diffused into the melt, then degassed and removed slag. The molten aluminum alloy was then held at 690 °C for 30 min before twin-roll casting. For the thin slab twin-roll casting process, the initial cast-rolling speed, melt temperature, roll gap, and cooling water pressure were 0.3−0.4 m/min, 690 °C, 20 mm, and 0.4 MPa, respectively.

After traditional TRC and FC-TRC experiments, the test samples were cut from the as-cast and annealed sheets along the rolling direction, respectively. Subsequently, hot rolling, cold rolling, intermediate annealing and heat treatment were performed on the rolling slabs, which parameters of solution and pre-aging treatment are 560 °C for 5 min and 150 °C for 8 min, respectively. In order to improve the accuracy of the mechanical property test, we prepared the samples on the 1 mm thick cold rolled sheets in three directions of 0°, 45°, and 90° with the rolling direction. The microstructure of the twin-roll casting strips were investigated under a Leica DMR optical microscope (OM, Leica, Solms, Germany). The inter-diffusion of alloy elements across the interfaces was studied on a JEOL JXA-8530F electro-probe micro (EPMA, Japan Electronics Co., Ltd, Tokyo, Japan) analyzer with an acceleration voltage of 20 kV and a sample current of 2 × 10^−8^ A. The interface morphology and composition of the phase were tested by a SHIMADZU SSX-550 scanning electron microscope (SEM, FEI, Hillsboro, OR, USA) equipped with energy dispersive spectrometer (EDS) scanning electron microscopy. The samples were prepared by mechanical grinding, etched by the Keller’s reagent from 20 s to 2 min. Tensile tests were performed using an AG-X100kN instrument (INSTRON, Canton, OH, USA) at a deformation speed of 1 mm/min.

To research the temperature field distribution in the cast-rolling zone, a thermo-mechanical model was built using the commercial software ANSYS (16.0, LMS, Leuven, Belgium) based process described above, three values of cast rolling speed (0.3 m/min, 0.35 m/min, and 0.4 m/min,) and two melt feeding methods were investigated. Several assumptions were made as follows: 

(1) The AA6022 melt is an incompressible Newtonian fluid and its flow in the calculation domain is laminar;

(2) The deformation of the roller is negligible and the cast rolling speed is constant; 

(3) The deformation heat of alloy is negligible compared with the heat of the melt; 

(4) No relative slip exists at the interfaces between the thin slab and roller;

(5) The solidified shell is closely contacted with the roll surface, and the partially solidified part of the roller contact surface is smaller convective heat transfer coefficients, while the other parts have larger convective heat transfer coefficients;

(6) The heat transfer method in the casting nozzle and the cast-rolling zone is heat conduction and thermal convection, and the convection heat transfer coefficients in the cast rolling process remains constant, ignoring radiation heat transfer;

Thermal conductivity (*K*), specific heat (*c*), density (*ρ*), and viscosity (*μ_e_*) of AA6022 aluminum alloy are shown in Table 1, respectively.

**Table 1:** Thermal physical properties of AA6022 aluminum alloy.

Parameters	Values
T_L_ (K)	920
T_S_ (K)	856
C (J/(kg k))	Figure 2a
ρ (kg/m^3^)	2352
μ_e_ (Pa s)	0.001 × 10^10*fs*^
K (W/(m k))	Figure 2b

The method of equivalent specific heat was used to account for the release of latent heat The latent heat was added to the specific heat of AA6022 alloy with Equation [8]:

$$c_{e}=c_{0}+\frac{L}{T_{L}-T_{S}}$$

This method is suitable for the treatment of alloys with wide solidification and can meet the requirements of most casting alloys. It is assumed that latent heat is released uniformly in practical calculation. The *c_e_* is equivalent specific heat; *c_0_* is the specific heat; *L* the latent heat of solidification; *T_L_* and *T_S_* the liquidus and solidus temperatures of AA6022 aluminum alloy. The values of *L*, *T_L_*, and *T_S_* are 3.76 × 10^5^ J/kg, 920 K, and 856 K, respectively.

## 3. Results and Discussions

### 3.1. Temperature Field under Different Conditions

**Figure 3:**
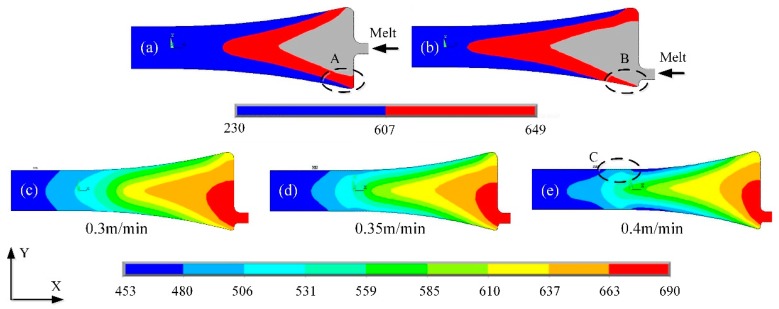
Simulation of temperature field in cast-rolling zone under different processing conditions: (**a**) aluminum melt is supplied in the middle of the nozzle, (**b**) aluminum melt is supplied in the lower part of the nozzle, (**c**–**e**) temperature field in cast-rolling zone under different cast-rolling speed.

The fabrication of AA6022 aluminum thin slab using a pilot-scale horizontal twin-roll caster was performed to investigate the relationship between the surface quality of the strip and the cast rolling speed. Figure 3a,b shows the effect of different feeding methods on the location of solid−liquid interface. We can see that the melt temperature of melt at zone A is too low to solidify in the cast rolling zone, which shows the cold laps defect on the surface of the slab, seriously affecting the mechanical properties of subsequent rolled sheet. The temperature at the zone B between the nozzle and the roller can be obviously increased by feeding aluminum melt on the lower side of the nozzle, thus avoiding the cold laps defect and improving the surface quality of the slab. 

When the other processing parameters are assumed to be constant, the cast-rolling speed *v* has a direct effect on the solidification profiles as shown in Figure 3c–e, the crystallization front are presented with the yellow wedge-shaped zones. With the increasing of cast-rolling speed, leads to a short contact time to the rollers, and solidification of the melt was delayed. The melt temperature and liquid fraction increases in the cast-rolling zone, heat loss of the melt through the cooling rollers decreases, results in a thin solid shell. As shown in zone C, heat line defect occurs when the cast-rolling speed increases to 0.4 m/min. Usually, surface quality increases with a decrease in rolling speed, the thin slab has sufficient contact time with the rollers. With the increases of separation rolling force, the surface of twin-roll cast strip becomes bright.

### 3.2. Optical Observations and Element Distribution

**Figure 4:**
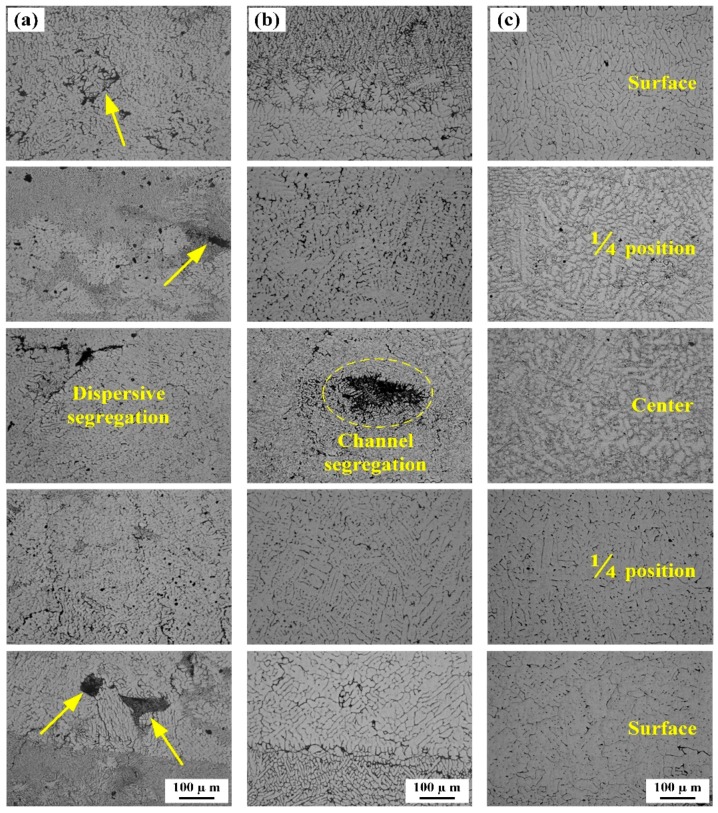
Microstructure of AA6022 alloy twin-roll cast strip under different processing conditions: (**a**) traditional twin-roll casting technology (TRC), (**b**) thin slab twin-roll casting technology (TS-TRC), and (**c**) Thin slab twin-roll casting with forced-cooling (FC-TRC).

**Figure 5:**
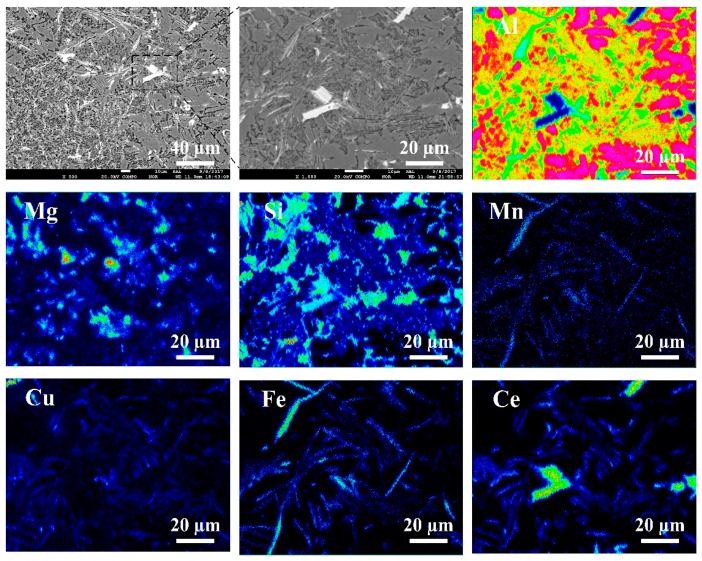
The electro-probe micro (EPMA) images of element distribution in the centerline segregation band region of twin-roll cast sheets.

As shown in Figure 4a,b, there are obvious macro-segregation defects and cracks in the traditional twin-roll casting method and thin slab twin-roll casting method, and the segregation defects at the surface and center regions of the strip, segregation defects black block-like region and black disk-like region. Further electron probe surface scanning experiments were examined at defect region to study the distribution of alloying elements (Figure 5).

The formation of the macro-segregation is obviously due to the separation rolling force applied during the twin-roll casting process. Solidification begins from the roll surface and dendrites grow against the direction of heat transfer, solute elements were enriched in the solid−liquid interface. Finally, the residual liquid melt enriched with solute elements was solidified by the water-cooled casting roller, forming non-equilibrium eutectic phases in the central area of twin-roll cast slab. This type of defect is more likely to occur under thin strip and high-speed twin-roll casting conditions.

As shown in Figure 4c, the centerline macro-segregation was significantly reduced with the application of forced-cooling twin-roll casting technology. The segregation is a cylindrical low melting point region. For thin slab twin-roll casting under high loads, the cast-rolling zone is divided into three regions: liquid zone, mushy zone, and solid zone. The deformation of the mushy zone results in liquid melt to be squeezed back into the crystallization front. When the liquid melt moved from a cold region to a hot region, the liquid melt will change its chemical composition, surrounding solid was re-melted to form the segregation. As shown in Figure 4c, massive centerline segregations are almost vanished after using forced-cooling twin-roll casting technology. According to Sengupta et al. [7], the forced cooling effect in the secondary cooling stage has an important influence on the solidification process. It is an important method to adjust the thin slab surface temperature, shell growth, and crystallization front position and to reduce the occurrence of segregations and side cracks.

As shown in Figure 5, the results of surface scanning maps, we found that the major alloying elements at segregation regions are Mg and Si. Compared with the two EPMA element maps the Si-rich zone also contains a certain amount of Mg element. That is to say, Mg_2_Si is not the only particle containing Si in the segregation regions. In the case of Mn and Cu, the elements are almost homogeneously distributed in the segregation regions. Theoretically, during the solidification process, with the advancing of the solid−liquid interface, low melting point compound were squeezed to the molten pool and solute elements at the solid−liquid interface increase gradually. In the ingots casting process, the segregation defect may be caused by the convection of the melt, but in twin-roll casting process, it may be due to the rolling force applied on the semi-solid region. Finally this defect can be continuously extended in the long rods of segregates in the longitudinal direction.

**Figure 6:**
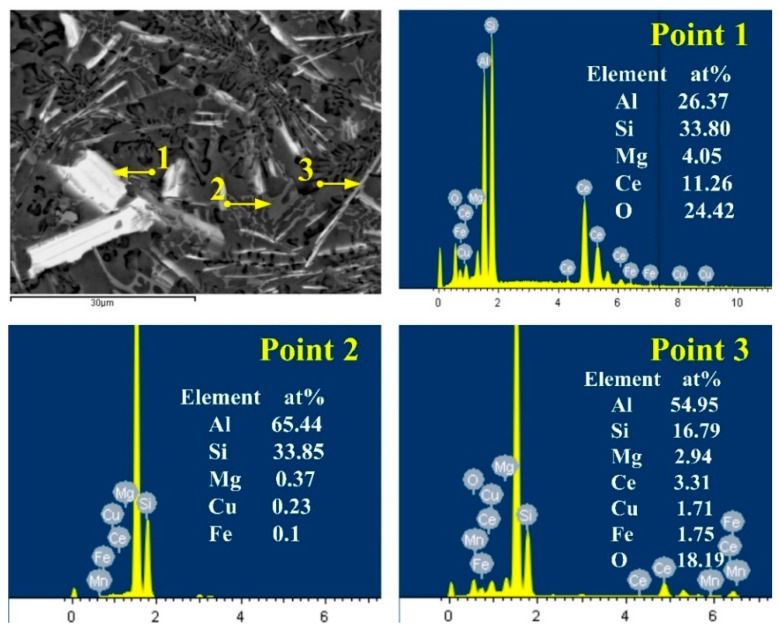
Energy dispersive spectrometer (EDS) point scanning results of point 1, point 2, and point 3 from the TRC alloy fabricated with the traditional twin-roll casting process.

In the above results, we found that centerline segregation of the twin-roll cast strip can be obviously reduced during the FC-TRC forced cooling stage. An EPMA experiment was performed on the segregation regions in order to investigate the distribution of alloying elements in AA6022 aluminum alloy, and a sequence of EDS images were shown in Figure 6, the results revealed that white lath-like region was mainly consist of 4.05% Mg, 33.8% Si, 11.26%Ce, and balance Al and O, and is defined as rare-earth intermetallic compound, The gray bone-like region consists of 33.85% Si, 65.44% Al, and balance of Mg and Cu, defined as Al–Si phase, The black ribbon-like region was consist of 9.97% O, 1.29% Mg, 6.37% Si, and balance of Al, Mn, and Cu can be highly supersaturated in aluminum matrix, so the atomic components of Cu and Mn are lower than 1%.

**Figure 7:**
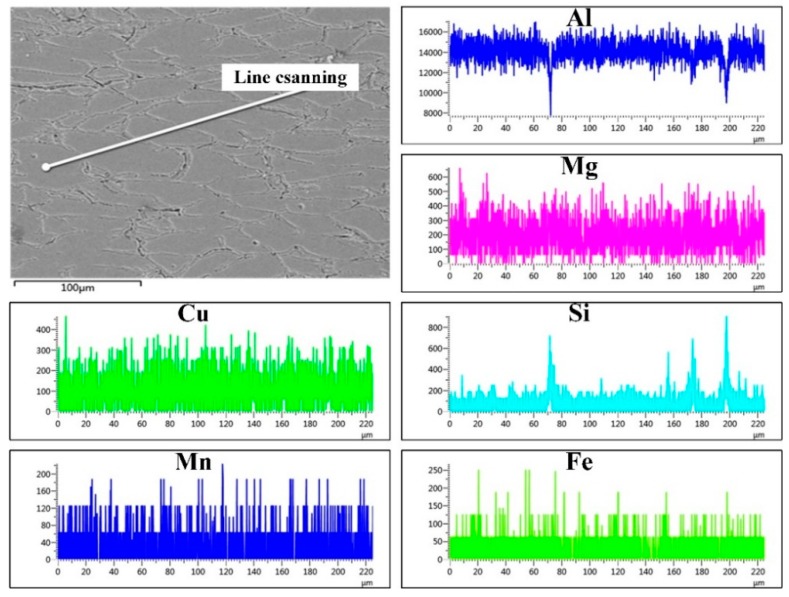
The distribution of different alloying elements in the segregation free region.

In the TRC sheet of aluminum alloy prepared by FC-TRC technology, we conducted a line scan at the region without segregation defects to study the distribution of alloy elements, and the results are shown in Figure 7. The gray rod-shaped phase at the grain boundary is mainly Si containing phase and a small part of iron containing phase. Within the grain, the content of Mg, Cu, and other alloy elements is relatively uniform, and there is no obvious large area element aggregation phenomenon. It shows that FC-TRC technology can effectively improve the distribution uniformity of alloy elements at the liquid−solid interface and reduce the number of non-equilibrium phases by changing the shape and depth of the mushy zone. 

### 3.3. Mechanism of Different Segregation Defects

Yun et al. have shown a graph where the strip thickness is plotted versus the specific rolling force (i.e., the total load applied divided by the strip width). If all the experimental results under different processing conditions are collected together, it is possible to build a convenient “defect limit diagram” to predict the relationship between processing technology and segregation defects [2,3,4].

**Figure 8:**
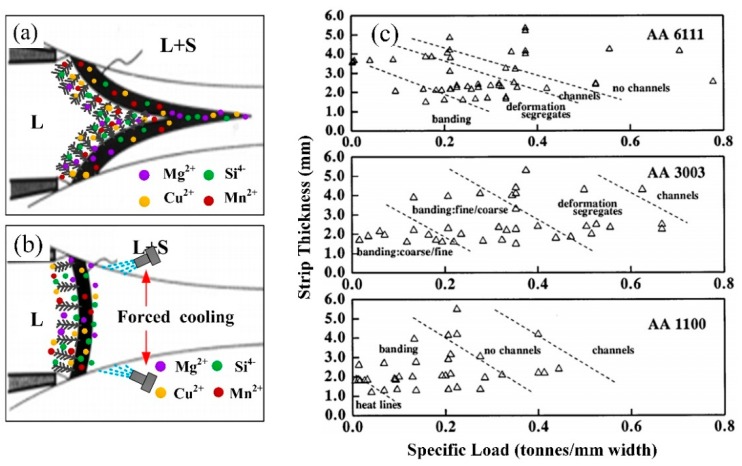
Formation mechanism of segregation defects. (**a**) Sharp crystallization front shape of traditional TRC, (**b**) smooth crystallization front shape of FC-TRC, (**c**) defect limit diagram of different alloys [24].

This graph is not a specific line between separation rolling force and strip thickness, but a trend can be shown. Defect-free material is found at the high-load, thick-slab right-hand corner. This is why we develop the thin slab twin-roll casting technology. The final thickness separating force can be divided into two areas: the defect free zone and segregates domain zone. As illustrated in Figure 8c, at thick gauges it is possible to fabricate strip without defects under all conditions except for thinner strip with lower separating force. If the thickness of the strip is reduced, the separating force of the strip should be increased correspondingly for the structure to remain free from centerline segregations. If the thickness reduced without increasing the separating force, segregation increases and the microstructure that is incompatible for subsequent processing and end product requirements. Detailed defect type reveals a gradual transition from centerline segregation through deformation segregation to banded structures. At even thinner gauges and high speed twin-roll casting condition, deformation segregates, and surface segregates predominate [2,24].

Nadella et al. reported that crystallization front depth is the most important parameters during TRC process, which exerts a dominant influence on the defects of both macro-segregation and hot cracking [25]. Theoretically, the crystallization front depth determines the quality in terms of macro-segregation, heat line, and hot crack tendency. As shown in Figure 8a, in the traditional twin-roll casting process, solidification begins from the roll surface direct to the center region, the melt enriched with solute elements is squeezed to the front of the solid−liquid interface. Finally, the centerline segregation was formed. The formation of the segregates is obviously due to a modification of the pressure applied on the strip during the twin-roll casting process. This phenomenon is particularly evident in aluminum alloys with wide solidification (the solidification of AA6022 is about 64 °C). It is well known that the severity of centerline macro-segregation increases with the crystallization front depth. With the separation rolling force increases, the heat transfer coefficient of the roller-strip is improved, solidification and deformation is so fast that the liquid melt cannot be squeezed from the solid phase, and the small droplets remain between the dendrites of the solid phase, forming a dispersive segregation.

There is an obvious relationship between the crystallization front depth with the cast-rolling speed and the twin-roll cast strip thickness. However, as shown in Figure 8b, in the novel FC-TRC process, with the application of forced-cooling device at the exit of the roll gap, crystallization front depth decrease and solid−liquid interface becomes smooth. Previous research have recently shown the parameters which can reduced the crystallization front depth will eliminate the centerline segregation, solute elements uniform distribution in the thickness direction of the thin slab. With the increase of strip thickness, the movement distance of solute elements increases [7]. Finally, the combined effect of two factors leads to the eliminating of centerline segregation defects.

### 3.4. Mechanical Properties

**Figure 9:**
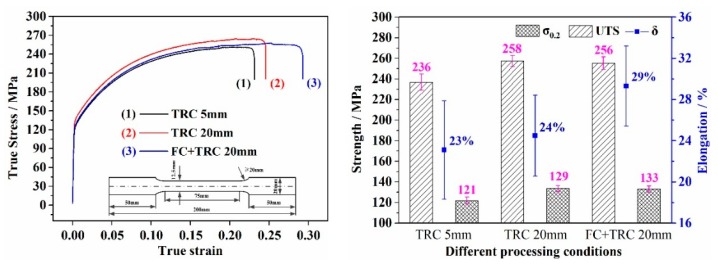
Microstructure and mechanical properties of strips of traditional TRC and FC-TRC conditions. (**a**) Strength of three strips and (**b**) elongation of three strips.

Figure 9 shows the tensile properties of strips with different processing conditions. The ultimate yield strength and tensile strength of strips solution heat treatment at 560 °C for 5 min and then immediately pre-aging heat treatment at 150 °C for 8 min are 121 MPa, 236 MPa, 129 MPa, 258 MPa, 133 MPa, and 256 MPa, with elongation of about 23%, 24%, and 29%, respectively.

The results indicated that the tensile strength of the FC-TRC strips nearly the same as the traditional TRC strips, but the elongation was the highest among the three simples. This is due to the absence of segregation defects. With the increase of reduction, more deformation energy for subsequent heat treatment process will be stored. The recrystallized microstructure becomes more homogeneous and fine resulting in an increasing in ductility.

### 3.5. Hardness and Electrical Conductivity

**Figure 10:**
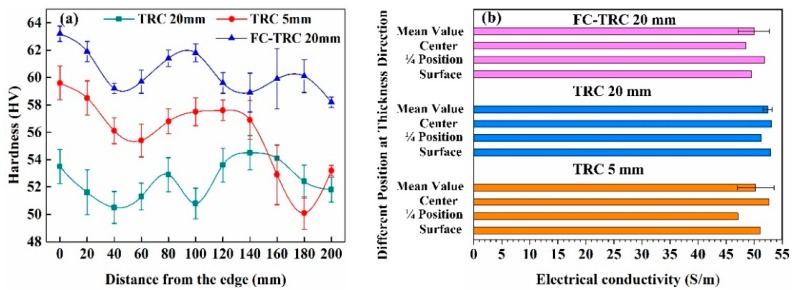
Hardness and electrical conductivity of TRC sheets of different processing conditions. (**a**) Hardness distribution along the width direction of TRC sheets and (**b**) conductivity distribution in thickness direction of TRC sheets.

In order to study the effect of different processing conditions on the solid-solution degree of alloy elements, we have examined the hardness in the width direction and the electrical conductivity in the thickness direction of TRC sheets. As shown in Figure 10a. 

Comparing the hardness values obtained under three groups of processing conditions, we can find that the hardness value of FC-TRC sheet is the highest, because the TRC sheet is encounter to secondary cooling at the moment when it comes out of the roll gap after the complete solidification at the kiss point, which reduces the precipitation quantity of the phase when the sheet is cooled down, thus increasing the solid solution degree of the alloy elements in the aluminum alloy matrix, resulting in the solid solution strengthening effect and improving the hardness.

In general, the higher the degree of alloying elements, the higher the strength and hardness, the opposite of the conductivity and plasticity. That is to say, the higher the degree that the alloying element is, the lower the conductivity is. This is because, after the formation of compounds between solute elements, the heterogeneous atoms, as solute elements, will cause lattice distortion as solvent elements, which will increase the distortion of the solvent lattice, the stronger the scattering effect on electrons, the greater the resistivity, and the lower the conductivity (Figure 10b). 

## 4. Conclusions

The simulation of the temperature field distribution, microstructure and mechanical properties of AA6022 aluminum alloy thin slab produced by a novel FC-TRC method were investigated. The key conclusions are summarized as follows:

(1) AA6022 aluminum alloys have successfully been produced into 20 mm thick thin slab at about 0.4 m/min with proper design of nozzle and graphite side dam, which can significantly improve the edge cooling effect, reduce the probability of cold laps defect, and the macro centerline segregation band.
(2) The novel FC-TRC method will change the crystallization front depth from sharp to smooth, increase the uniformity of the alloy elements in the thickness direction of the thin slab. This greatly reduces the tendency of alloying elements to concentrate at the center region of the strip which resulted in an increasing in ductility properties.
(3) After further cold rolling and the heat treatment process, the ultimate yield strength and tensile strengths of strips are 121 MPa, 236 MPa, 129 MPa, 258 MPa, 133 MPa, and 256 MPa, with elongation of about 23%, 24%, and 29%, respectively. Compared to the traditional TRC method, the elongation of FC-TRC increased greatly but the tensile strength was nearly the same.
(4) The effect of the forced cooling method can significantly improve the solid solution of alloy elements, increase the hardness and decrease the conductivity. The average hardness increased from 52.45 HV of traditional TRC to 60.35 HV of FC-TRC, and the average conductivity decreased from 50.27 HV of traditional TRC to 49.95 HV of FC-TRC.

## Figures and Tables

**Figure 2:**
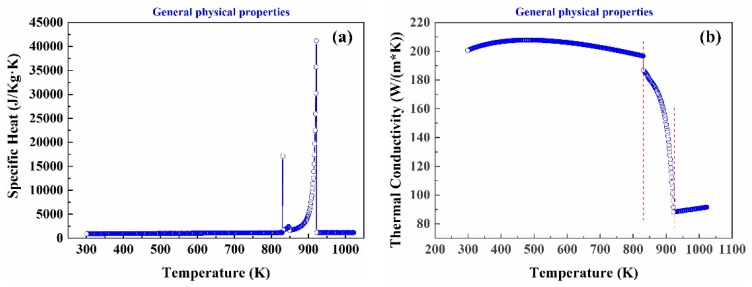
Thermal parameters of aluminum alloy AA6022 (**a**) specific heat (**b**) thermal conductivity.
